# Retraction Note: Zingiber officinale rhizome extracts mediated ni nanoparticles and its promising biomedical and environmental applications

**DOI:** 10.1186/s12906-026-05394-3

**Published:** 2026-05-05

**Authors:** Abdullah, Tahir Hussain, Shah Faisal, Muhammad Rizwan, Mervt M. Almostafa, Nancy S. Younis, Galal Yahya

**Affiliations:** 1 Department of Physical Chemistry and Technology of Polymers, Silesian University of Technology, M. Strzody 9, Gliwice, 44-100 Poland; 2 Joint Doctoral School, Silesian University of Technology, Academika 2a, Gliwice, 44- 100 Poland; 3 Department of Microbiology, Abdul Wali Khan University Mardan, Mardan, Khyber Pakhtunkhwa 23200 Pakistan; 4 Institube of Biotechnology and Microbiology, Bacha Khan University, Charsadda, Khyber Pakhtunkhwa 24460 Pakistan; 5 Center for Biotechnology and Microbiology, University of Swat, Swat, Khyber Pakhtunkhwa 19000 Pakistan; 6 Department of Chemistry, College of Science, King Faisal University, Alhofuf, Al-Ahsa 31982 Saudi Arabia; 7 Department of Pharmaceutical Sciences, College of Clinical Pharmacy, King Faisal University, Alhofuf, Al-Ahsa 31982 Saudi Arabia; 8 Department of Microbiology and Immunology, Faculty of Pharmacy, Zagazig University, Zagazig, Al Sharqia 44519 Egypt

**Retraction Note: BMC Complementary Medicine and Therapies 23, 349** **(2023)**

**https://doi.org/10.1186/s12906-023-04182-7**

The Lead Editors have retracted this article. Concerns were raised regarding Fig. 3b, as multiple regions within the image display similar particle arrangements. The authors were unable to provide verifiable primary raw SEM data corresponding to the published figure. The Lead Editors therefore no longer have confidence in the reliability of the data reported in the article.

Author Abdullah has stated on behalf of all authors that they do not agree to this retraction.

